# Medicine and the Public: The 1962 Report of the Royal College of Physicians and the New Public Health

**DOI:** 10.1353/bhm.2007.0000

**Published:** 2007

**Authors:** Virginia Berridge

**Keywords:** smoking, public health, media, evidence-based medicine, social medicine, permissive society, consumerism*[Other P-286]*

## Abstract

The 1962 report of the Royal College of Physicians on smoking was a significant event in the history of smoking. Its significance was, however, more than smoking-specific: the RCP committee's appointment, its membership, its work, and the manner of its publication signified the changes within social medicine, and within the medical profession more generally, in postwar Britain. Doctors assumed the right to speak to the public and to government on matters of individual health, and a new risk-based public health was in the process of formation. A public health “policy community” formed, and governments began to assume responsibility for advising the public on health matters. The use of research in the report, and of social research in response to it, was important in the emergence of evidence-based medicine within public health. The paper argues for greater attention to the change in public health epitomized by the report in current debates on the concept of the 1960s “permissive society.” It was the harbinger of a new style of “coercive permissiveness” in health.

In April 1963, D. Kelly wrote to the British Ministry of Health about an idea he had had in his head for quite a while about antismoking publicity. After discussion with a German doctor friend, he suggested: “A rhyming poster might work … . ‘THE MODERN BLOKE—DOESN'T SMOKE’… . The ladies are less of a problem—but a growing one. What about ‘CONTEMPORARY HAGS ABHOR FAGS’ with a similar illustration of modern witches refusing temptation.”^1^ Another correspondent, K. Norman Reynolds, had written in the previous month. He enclosed a poster he had originally designed for a competition, but was, “alas,” “too late in entering it”: “The word ‘Cancer’ is spelt in cork tipped cigarettes, which gets across a point as well as adding to the eye appeal. This unfortunately hasn't come out in this print.”^2^ In the early 1960s, the Ministry was also the recipient of “puffng poems” and drawings, the results of a National Society of Non Smokers essay competition for children. Antismoking ideas poured in from members of the public.

These suggestions, now yellowing in their folders in the National Archives, are testimony to the change that occurred in the 1960s and 1970s in public health, and, indeed, in the relationship between medicine and society more generally. For the talk of posters and homemade publicity efforts represented the last gasp of an older tradition of public health and of public education, but also looked toward new developments. A new era of mass-media education and health consciousness of individual risk was dawning. Both David Armstrong and Mark Harrison have seen the war years as important for the rise of health education—either as Armstrong's “medicine of the social”^3^ promoted by the wartime need to know, or Harrison's argument that wartime health education in the army promoted a new mood of citizenship and responsibility.^4^ The late 1950s and early 1960s saw a reorientation of that wartime stance on the part of government: citizens who would act responsibly if given “the facts” were replaced by consumers of harmful goods or substances who needed to be persuaded about risk. In the early 1960s, medicine began to modernize itself, repositioning itself in relation to government, and to society and “the public.” I argue here that the report on smoking published by the Royal College of Physicians in 1962, *Smoking and Health*, was a key stage[Other P-287] on the road to the new modernized and mediatized medicine and public health.

The repositioning and its implications are central to two areas of historical debate—to reassessments of “the permissive society” of the 1960s, and to the historiography of public health. For the former, commentators such as the health-policy analyst Howard Glennerster have noted that a new social-policy agenda was emergent in Britain in the 1960s and 1970s, which removed criminal sanctions in regard to abortion and sexuality and was hostile to state intervention in such matters.^5^ But others have noted that criminal forms of regulation were replaced by medical ones, and that “permissiveness” in sexuality was dependent on new forms of medical surveillance.^6^ The “myth” of 1960s permissiveness has come under scrutiny in a wider range of more recent work.^7^ The revision has not, however, discussed the changes in public health in that decade that are considered in this paper. I seek to argue that the 1962 report and the changes it helped to usher in in public health in the 1960s, and especially in the 1970s, embodied the contradictions in the concept of permissiveness: on the one hand, health became a matter of individual responsibility; but that individual responsibility lay within a new framework of governmental intervention in individual behavior—what is termed here “coercive permissiveness.”

The historiography of British public health is beginning to take account of the postwar changes in the ideology and outlook of public health.^8^ Most attention has been focused on the organizational and professional changes that saw the Medical Offcer of Health (MOH) lose his local government “empire” in the early 1970s and reemerge as the “community physician” located within the National Health Service (NHS). Medical public health professionals have been criticized in Jane Lewis's work for the failure to develop a distinctive ideology for public health, and for their tendency to defne the role of public health around whatever tasks they undertook at the time.^9^ Journalist doctors like James Le Fanu and Michael[Other P-288] Fitzpatrick have discussed the subsequent history of public health. They have criticized the rise of a new “health tyranny” through health promotion, but they have focused primarily on later events and key issues like the government health-education campaigns on AIDS in the 1980s.^10^

I argue in this paper that both sets of historical debates need to incorporate consideration of the 1962 Royal College report and the rise of an ethos of public health not tied to health services, to MOsH, or to community physicians. The 1962 report was highly signifcant for the history of smoking policy, but its signifcance was also a wider one. *First, it signifed a new willingness on the part of medicine to speak to the public, and to use the media to do so.* The media became central to public health. Doctors reoriented their role so that they spoke to the public, not just to the rest of the profession. The role of the media also became central to debates within public health: on the one hand, mass-media campaigns were increasingly important as a strategy and began to focus on the role of individual risks to health, to urge the reformation of behavior; on the other, the control and even prohibition of advertising deemed detrimental to health was to become an important public health strategy. The wartime and immediate postwar emphasis on responsibility and citizenship gave way to an emphasis on propaganda and persuasion using consumerist techniques. *Second, it marked the emergence of a “policy community” around public health, linking civil servants within government with medical experts outside*. This model of health policymaking was, with variations, to dominate the process of British health-policy formation into the twenty-frst century. British government carried on a policy-balancing act in which the role of insider/outsider organizations and formal interconnections with scientifc expertise were increasingly important. *Third, it emphasized the role of individual behavior, legitimated through population-based epidemiology, as the dominant focus of public health endeavor in postwar Britain*. The report gave public signifcance to a new type of public health and to different scientifc ways of studying it. The new epidemiology of the 1950s and the new focus on the risk of chronic disease were translated into a wider public and policy agenda. *Fourth, it stimulated new attitudes on the part of government regarding its relation to the public on matters of health, and a heightened signifcance for research-based surveillance*. Medicine and consumerism were allied through a focus on the role of the individual in society, and through a new emphasis on[Other P-289] individual persuasion. At the same time, research and the social survey began to outline a new view of “the public” and to establish a relationship between medicine and the social sciences, one that built on the alliances within social medicine but also turned them in a new consumerist direction. It was part of the rise of evidence-based medicine.

The report therefore signifed a new style and outlook for public health that was emergent at around the same time as the organizational and professional changes, but was, in many respects, separate from them. The smoking activists were not MOsH or even the new community physicians: a new public “public health” was emerging, distinct from the profession and its service role. This was research- and “evidence-based,” using the social sciences as technical tools. Such developments also invite refection about the nature of the permissiveness of the 1960s and the roots of the “health tyranny” that the journalists have criticized. The health discussions of the 1960s were marked by contradictory tendencies: in one sense, by the very antithesis of permissiveness; in the other, by a new style of “coercive permissiveness” in health.

## The Prehistory of Smoking and Lung Cancer

The early history of the smoking-and-lung-cancer connection is well known and has been recounted in a number of different works.^11^ Concern was roused by the gradual increase in the incidence of cancer; by a change in the balance of the sexes, toward men; and by the increasingly important role of lung cancer. The greatest increase in lung cancer came in males over forty-five, where the incidence increased sixfold between 1930 and 1945. At first it was thought that these changes might be due to improved diagnosis and better recording and registration. Work carried out by Sir Ernest Kennaway in the 1930s and published in 1947, a detailed examination of postmortem certifcates, helped eliminate occupational and environmental factors. Kennaway pointed to a connection with cigarette smoking, but his work, based on statistical correlations, carried little weight because of the perceived lack of legitimacy of this mode of explanation at the time. Laboratory studies tended to support the connection. Research had also been undertaken before the war in Nazi Germany, and by the American biometrician Raymond Pearl, for the insurance indus-[Other P-290] try.^12^ The issue became more urgent after the war, and discussions between the Ministry of Health and the Medical Research Council (MRC) led to the council's convening an informal conference on cancer of the lung in February 1947. The MRC agreed to initiate a large-scale statistical study of the past smoking habits of those with cancer of the lung, and of two control groups. This was the origin of the work carried out in the Statistical Research Unit at the London School of Hygiene and Tropical Medicine (LSHTM) by Professor Austin Bradford Hill and Dr. Richard Doll. The results, published in the *British Medical Journal* in 1950, concluded that there was a “real association” between carcinoma of the lung and smoking, and that smoking was a factor, and an important one, in the production of lung cancer.^13^ Work by Ernest L. Wynder and Evarts A. Graham in the United States had come to similar conclusions.^14^ Later prospective studies carried out by Doll and Bradford Hill and by Edward Cuyler Hammond and Daniel Horn in the United States appeared to implicate cigarette smoking even further.^15^

Charles Webster has shown in detail how the issue fared over the next seven years.^16^ A written parliamentary answer from Ian Macleod as Conservative minister of health in February 1954 accepted that there was a connection, but that it was not a simple one.^17^ When the MRC issued its own report on smoking and lung cancer in June 1957, the Ministry of Health adopted the argument more fully. The parliamentary secretary to the Ministry of Health (MH) for the frst time on 27 June 1957 expressed unambiguous support for the conclusions reached by Doll and Hill in[Other P-291] 1950. Webster locates this sequence of events in the machinations of the powerful and complex advisory machinery that stood between the MRC and the MH: the main advisory body was the Cancer and Radiotherapy Standing Advisory Committee, reporting to the Central Health Services Council, which in turn advised the Ministry of Health. Horace Joules of the Central Middlesex Hospital, a member of both bodies, was the only person within the advisory-committee machinery consistently to press the issue; Paolo Palladino has recently related his stance to a continuing Christian Socialist tradition.^18^ The initial governmental response focused on a Ministry of Health circular encouraging local authorities to develop health-education campaigns on smoking. Further action under the Labour government of the 1960s saw the banning of cigarette advertisements on television in 1965, and attempts by the Labour minister of health, the GP Kenneth Robinson, to introduce legislation to ban cigarette coupon schemes and to limit other forms of advertising. Health warnings on cigarette packets appeared in 1971. This was the “end of the beginning” of the frst phase of the policy response.

The Doll/Hill research of the 1950s had impacted upon a fuid policy situation in that decade in which the governmental response was conditioned by a number of factors, not all of them directly smoking-related.^19^ The economic importance of smoking to the exchequer was considerable, and the tobacco industry was a valued partner of government, building on formal controls that had operated during wartime. But also in play were changes in the nature and role of public health; the role of air pollution as a contentious political issue; the contested nature of the evidence; the central governmental politics of health education; and the general culture of smoking, with its electoral implications. The last of these was a crucial issue for politicians: did governments have the right to tell the public what to do about a culturally sanctioned, acceptable habit that might possibly lead to disease many years hence? and, what would this mean in terms of political popularity?

It was also a crucial issue for public health. The British social-medicine ideology of the 1930s and 1940s had stressed the need for a holistic vision of medicine, and key research papers had talked of occupation and of class as crucial dynamics. But, as Dorothy Porter's work has shown, the[Other P-292] ethos of social medicine was changing in the 1950s, with a new emphasis on the role of individual psychology and of issues such as “stress.”^20^ Social medicine was reorienting itself to a focus on chronic-disease epidemiology, of which the smoking work formed part, and which laid stress on the role of the individual. Central to this reorientation was the classic text by the social-medicine pioneer Jerry Morris, *Uses of Epidemiology*, published in 1957.^21^ Morris's paper on the impact of exercise on heart disease tellingly compared the rates of heart disease of sedentary bus drivers with those of active conductors, combining the occupation and class emphasis of 1940s social medicine with the emergent interest in individual behavior.^22^ Increasingly, too, such interests were looking outside the confnes of the closed medical world and reaching out to a new engagement with “the public,” an activity that previous bans on medical advertising had prevented.^23^ The involvement of Charles Fletcher (who was a pioneer of the new media- and public-focused developments) and of Morris in the 1962 Royal College committee was thus highly signifcant.

## The Origins and Membership of the Royal College Committee

Nevertheless, the Royal College of Physicians (RCP) was not the most obvious body to produce a report on the link between smoking and lung cancer, and it had already turned down the opportunity once. In November 1956, Francis Avery Jones, a gastroenterologist from the Central Middlesex Hospital with whom Doll had originally worked, wrote to the president of the College, Lord Brain, urging that the College put out a statement on the effect of smoking on health, “with particular reference to the rising generation.”^24^ Brain—a shy, reserved man—took a month to reply, only to turn the proposal down. The reasons for his refusal were, in their dislike of giving public advice, typical of the profession's attitude at the time:[Other P-293]

The work of Richard Doll and Bradford Hill has received very wide publicity and must be known, I should imagine, to every doctor in the country, so it is diffcult to see that the College could add anything to the knowledge of the existing facts. If we go beyond facts, to the question of the giving of advice to the public as to what action they should take in the light of the facts, I doubt very much whether that should be a function of the College.^25^

Subsequently, the College's attitude changed. In 1957 Robert Platt was elected president as successor to Brain. Platt had a modernizing agenda for the profession, which smoking ftted admirably. He was first approached on the subject of smoking by Charles Fletcher, frst director of the MRC's pneumoconiosis research unit in Cardiff, who at the time of his approach was working as a respiratory physician in the department of medicine at Hammersmith Hospital. Fletcher had been invited to lunch by the deputy chief medical officer, George Godber, who was frustrated by the lack of activity within his Ministry, and the two had agreed to sound out Platt about taking on the smoking issue.^26^ Godber was a member of the RCP's Council and a close friend of Platt. Avery Jones also heard what was afoot and wrote again in January 1959 to urge the Royal College to action. The first informal meeting was held on 16 February 1959, and in April the Comitia of the College agreed that a committee should be formed “to report on smoking and atmospheric pollution in relation to carcinoma of the lung and other illnesses”; the first formal meeting was held at the College on 15 July 1959 at 5 p.m.^27^

This sequence of events was illustrative of wider changes in postwar medicine. Smoking was a chance for the Royal College to position itself in relation to new agendas emerging in health. Medical interest had been in occupational health and in the environment and disease—symbolized by Fletcher's own previous occupational work on miners' lung disease and his interest in air pollution and chest disease—but these interests were giving place to a new focus on chronic diseases of the individual brought on by habits like smoking. The networks that operated in this instance were also signifcant for the future: Godber was a graduate of the London School of Hygiene and Tropical Medicine (LSHTM), the foremost public health school. His position as a medical civil servant in[Other P-294] the Ministry of Health, but working closely to a health agenda with medical and health interests outside, was illustrative of the emergence of the “policy community”—the term used by political scientists to analyze how policymaking interests work, with interests within government forming alliances with those outside. These alliances were to become important in the making of postwar health policy, particularly in relation to the medical profession.^28^ British civil servants are neutral and nonpolitical fgures who do not change when governments change; the role of the Chief Medical Offcer in government was as a neutral adviser, and the Ministry of Health had a twin-track bureaucratic organization with both specialist medical and generalist civil servants.^29^ The links between the former group of civil servants and outside medical interests were important in this instance and for other issues in postwar health policymaking. Platt's interests in medical modernization extended widely-he was a leading fgure behind the subsequent Todd committee on medical education in 1968, and was also important in new moves around genetic disease in Manchester.^30^ The creation of the College committee symbolized the changing role of medicine.

The membership of the committee was also symbolic. It was decided informally through the networks of British social medicine, with Fletcher and Platt in leading roles and Godber behind the scenes. Platt was in the chair, but Fletcher as its secretary was the moving spirit of its work. Fletcher was the son of Walter Morley Fletcher, former secretary of the MRC; he had “all the confidence of the Old Etonian” and impeccable connections in medical circles, but also a social conscience and a commitment to communicating with the public through the media.^31^ In 1958 his series *Your Life in Their Hands*, showing surgical procedures on television, had caused huge controversy. The series had been part of developments in medical broadcasting. It had originated in a set of programs called *Thursday Clinic* transmitted in 1954 and 1956, consisting of outside broadcasts from St. Mary's hospital in Paddington. The work of NHS hospitals had been seen in earlier programs such as *Matters of Life and Death*[Other P-295] (1951) and *Matters of Medicine* (1952), and medical procedures were also shown in *The Hurt Mind* (1957), which dealt with new developments in the treatment of mental illness and in which Fletcher was also involved.^32^ Such programs, and the media controversy over cases of conjoined twins in the 1950s, had begun the reordering of relationships around medical confidentiality which had up until then been a constraining issue for public depictions of medicine.^33^

Fletcher was a leader of these developments—but other members of the committee were also closely involved in the new relationships between medicine and the media: Dr. Guy Scadding had appeared in *Matters of Medicine* explaining the complexities of the interactions between lung cancer, smoking, and air pollution. Jerry Morris, of the MRC-funded Social Medicine Unit, had given radio talks, including one in 1955 whose content foreshadowed the new developments in public health that the RCP committee came to symbolize:

We are dealing with a different social situation. The nineteenth-century epidemics, bred in poverty and malnutrition, arose from the failures of the social system… . But coronary thrombosis … with its origins apparently in high living standards … seems to be arising from what we regard as successes of the social system… . It is becoming clear that in the modification of personal behaviour, of diet, smoking, physical exercise and the rest, which look like providing at any rate part of the answer, the responsibility of the individual for his own health will be far greater than formerly. It will not be possible to impose from without (as drains were built) the new norms of behaviour better serving the needs of middle and old age. They will only come about in a new kind of partnership between community and individual.^34^

Morris's advocacy of media and advertising initiatives on the committee was strong and a continuing strand in his long career; in 2000 at his ninetieth-birthday conference at LSHTM, the leading epidemiologist Michael Marmot remarked that “Jerry has always told me that I should watch more television rather than less.”^35^ Others on the committee, like[Other P-296] Avery Jones, the gastroenterologist who had originally suggested action to the RCP, symbolized the new medical interest in smoking and chronic disease, while the presence of Sir Aubrey Lewis of the Institute of Psychiatry indicated the role that psychological insights were to play in the new developments in public health. The committee subsequently added Dr. N. C. Oswald to its number; he was a smoker, and all the rest of the committee were by then nonsmokers. The committee also consulted experts including Richard Doll and Alexander Haddow of the Chester Beatty Institute, and Godber was also available, although not a member of the committee. In an interview, Morris remembered that they had tried to involve a Medical Offcer of Health with an interest in smoking, but could not fnd one.^36^ That comment was indicative of the gulf between academic and practice-based public health. The committee's membership emphasized the networks that were beginning to coalesce around the new risk-based public health. It also symbolized an alliance between Fletcher's prestigious medical connections and the clinicians and social-medicine people and epidemiologists, who had lower status within the profession.^37^ (They had originally been located at the Central Middlesex Hospital in Willesden, a North London suburb; as a former local government hospital under the aegis of the London County Council, this had much lower status than the prestigious London teaching hospitals.)

## The Work of the Committee

The work of the committee proceeded through nine meetings between 1959 and 1961, often with long gaps between them. Much was done outside the committee, with members preparing papers and gathering evidence. Early on it made two key decisions that emphasized the new directions in public health. First, it decided to speak directly to the public rather than to the profession. The minutes of the fourth meeting, on 17 March 1960, recorded that a discussion was opened by the president on how the report should be presented: “The usual College report had limited circulation among the medical profession”; therefore,[Other P-297]

it was agreed that the Committee's report should have more publicity and a wider circulation than the usual College reports. It could not advise government on any course of action, but it could suggest lines of action.^38^

Second, it disposed of the air-pollution connection. Although the Comitia of the Royal College had wanted a report that combined discussion of both issues, the committee decided not to produce this:

It was agreed that the evidence would be of an entirely different quality and nature. It was pointed out that individuals could avoid the dangers of smoking but not those of pollution. It was also thought that a section on atmospheric pollution within the main report might detract from the main arguments on smoking and lung cancer.^39^

The committee did eventually produce a separate report on air pollution, but this was not published until the early 1970s and without much sense of urgency. The committee recognized that the issue of smoking and lung cancer was a much more clear-cut case where individual action could be stressed. On both counts, the committee was moving toward a concept of health that focused more clearly on individual responsibility and that could be expressed through appeals to the public rather than to the profession.

The areas of the committee's work were divided between members according to their own interests, so memoranda appeared through the meetings on diseases of the lung, on the chemistry and pharmacology of smoking, on smoking and the gastrointestinal tract. Interest in consumer issues, in advertising and the media, and in what the public thought and how it could be infuenced formed signifcant threads in the discussions. Early on, Aubrey Lewis produced a paper on the psychological aspects of smoking that pointed out the lack of evidence that health education could discourage inveterate smokers; school-based prevention might be more effective, but there was little information on projects that had been undertaken.

Lack of information on these newer strategies and aspects of health interest was a theme throughout the work of the committee. Economic issues and consideration of the role of the media were to be of growing importance within the new ideology of public health—but in the late 1950s and early 1960s the profession of health economics was still in the future, and it was the social-medicine interests that took up the economic and media issues. It was the social-medicine pioneer Jerry Morris who was[Other P-298] active throughout the life of the committee in investigating consumer expenditure and the role of tobacco advertising. His work showed the expanding importance of television advertising in the situation; the committee therefore pressed for an offcial survey of smoking habits in children and inquired into advertising controls on television.^40^ Morris also brought the issue of coronary heart disease (CHD) into the committee's discussions, infuenced by the early publications of the Framingham study that had investigated CHD in the United States.^41^

Fletcher drew together the final report, making it accessible to the lay public, but clearly other members of the committee played an important role; Morris's work was particularly signifcant for the public, media, and consumerist emphasis. It was agreed that the report should include a section on the use of advertising against smoking: “modern methods should be employed to combat modern methods.”^42^ Public health at this stage had close relationships with the British tobacco industry. Imperial Tobacco, the main industry organization, was seen by some public health interests and by government as a partner in a shared enterprise to reduce harm from smoking.^43^ Geoffrey Todd, the Imperial Company's lead statistician, and others from the Tobacco Manufacturers Standing Committee provided information and statistics for the final report; the report was also shown informally to the Tobacco Manufacturers Standing Committee before publication.^44^

The report was finally published in March 1962. Its form, content, and presentation were signifcant. Surveying the history of smoking, the chemistry and pharmacology of tobacco smoke, and the latest scientifc evidence about the relationship with cancer, gastrointestinal diseases, lung disease, and coronary heart disease, as well as the psychology of smoking, it laid out a possible seven-point agenda for governmental action. Five of the seven points were consumerist and media oriented: public education, restrictions on sale to children, restriction of tobacco advertising, tax increases (and perhaps differential taxation for less harmful pipes and cigars), and information on the tar and nicotine content of cigarettes. Only two points came from different traditions: the environmentalism[Other P-299] of restrictions on smoking in public places, and the “medical model” of antismoking clinics.^45^ The agenda for government thus largely dropped action on the environment (air pollution) and gave full rein to the new appeal to the public, to economic and consumerist trends.

## The 1962 Report and the Appeal to the Public

The manner of the report's presentation and publication symbolized this. The College hired a public relations consultant, Roger Braban, to manage the launch of the report, and held its first-ever press conference. Braban recalled:

I came in as PR consultant to the RCP a few months before the smoking report—they had never used a professional launch … then they got a taste for it and used it for every report… . I spent a lot of time in finding the right team… . the President and Charles Fletcher, he was a popular figure with the media… . I timed it so that Ministers had the report before it was published—they feel they're party to something.^46^

Charles Fletcher later gave a favor of that first press conference:

On the day before publication a press conference was held at the College and it was crowded. Many questions were asked. When one reporter quoted that the annual risk of lung cancer in heavy smokers aged 55 was only one in 23, the President asked him if he would fly with an airline only one in 23 of whose planes crashed he agreed he would not. Next day there was fortunately no big news and the report got major headlines, Robert Platt on the BBC and I was interviewed on ITV.^47^

The report was also marked by a special program on *Panorama*, the flagship TV vehicle for current affairs, which went out on television on 12 March, just after the publication of the report. Fronted by the commentator Richard Dimbleby, the program interviewed scientists (mostly laboratory based) and members of the public about their response, and about giving up smoking. The centerpiece of the program was an interview by the presenter Robert Kee with John Partridge (chairman of the Tobacco Manufacturers Standing Committee) and Sir Robert Platt. The standoff between the two, with Platt robustly interrupting Partridge's defense of the industry, made good television:[Other P-300]

KEE: Mr Partridge, would you agree that we must stop young people smoking?

PARTRIDGE: No I would not, and let me, just while I can, take up one point that Sir Robert made just now. The *Observer* had no right to make that remark in its editorial yesterday, Sir Robert, and nor, with respect, have you.

(INTERRUPTION)… about only a tobacco manufacturer could deny this.

KEE: Well that is the position we have here now isn't it?

PARTRIDGE: It is so, but the implication is some dishonest approach to this problem, and that is not well founded.

(INTERRUPTION)… May I just finish here… ^48^

This was unusual television for the time, but it was a portent of the future “mediatization” of health issues and the premium it put on conflict and opposition.

The report was popular with the public. Originally the College had wanted only 5,000 copies printed, and when Fletcher insisted on double that number, it had required the committee to pay for any copies that were unsold. But the report sold out within a few days, and a second printing was needed. It had sold more than 33,000 copies in the United Kingdom by the autumn of 1963, and more than 50,000 in the United States.^49^ It was followed the next year by Fletcher's “Penguin Special” volume, *Common Sense about Smoking*, which symbolically linked the medical evidence with a chapter on economic effects and others on social implications and how to stop. Here was a further attempt to appeal to the public, which brought together what was to become a common combination in public health: a review of the science coupled with a self–help guide to individual reformation.^50^

## The Work of the Cabinet Committee on Smoking

What was the government's response? Governments of the period have often been criticized for inadequate responses, reliant on health education rather than more stringent measures of control. But the choice of health education as the main response, and the change in the nature of that education, was significant. Just as medicine in this period was reorienting toward a public advice role, so too can we see governments of both[Other P-301] political persuasions, Labour and Conservative, moving toward a new view of their role in relation to the population and health matters, in line with the changed profle of disease. Governments began to assume a new duty to advise and warn about health risk, to persuade their citizens rather than to assume that a sense of public duty inherent in the population would lead them to make up their own minds. Politicians remained concerned about the electoral implications of such a stance— but their opposition to intervention in such matters was in decline by the end of the 1960s.

Governments began to seek to infuence the health habits of those whom they governed. To do this, they also began actively to seek out information about them—about the beliefs and habits of normal populations, and about their health—through surveys and other research mechanisms, a development that paralleled the increased emphasis on populations within chronic-disease epidemiology. This was an important change that again built on the wartime social surveys and gave a new role to research and also to quantitative social science.^51^ The social science disciplines assumed heightened technocratic significance in relation to these developments. The 1962 report was an important catalyst for the “evidence-based” tendency within the new public health.

Let us look at how these responses developed in the 1960s. The main vehicle for governmental response to the report was the cabinet committee on smoking, which reported to the main cabinet. Cabinet committees had been briefly formed in the 1950s at the time of the various parliamentary statements, and had been chaired by the home secretary of the day. R. A. Butler, as home secretary, chaired the first meeting of the latest committee; but Harold Macmillan, the prime minister, did not want Butler in this role and Lord Hailsham, lord president of the Council, took over. The ministerial committee was paralleled by one of officials, which did the detailed work.^52 ^The officials moved swiftly: the first meeting of their committee was on 23 March, two further meetings followed, and a draft report was ready to go to the lord president by the middle of April.^53^ The report, preceded by a flurry of activity in the relevant departments, was relatively anodyne, placing its reliance on health education and on voluntary agreements for advertising. The officials came down against[Other P-302] differential taxation (taxation graded according to the harm occasioned by the product—so that pipes and cigars, thought to be less harmful, would attract lower tax rates than those for cigarettes) and the taxation option in general: taxation, it was argued, would penalize the poor, raise the cost of living, and have a serious effect on producer economies in the empire such as Rhodesia. This view reflected the belief that more-restrictive action could not be sustained without major change in public attitudes to smoking. Research in Edinburgh and the government's own pilot survey of public attitudes to smoking through the Central Office of Information (COI) had confirmed that most people knew about the link between smoking and lung cancer, but their views on why smoking was harmful to health were different from those of the scientists: the public view of smoking stressed the environmental-nuisance aspects rather than the risk-based epidemiology.^54^

The politicians did not agree on the sales-to-children issue, nor on differential taxation. The Treasury fought strongly against the latter, and ultimately the committee could not agree. In the event, education and voluntarism were the keynotes of the response, and the committee decided not to make a statement. As Hailsham told Macmillan, a small publicity campaign would not be welcomed, and in any case interest had abated for the present. He proposed to set up the machinery and start the campaign, perhaps issuing a statement later on. A meeting with the manufacturers might also result in an agreement to apply the TV restrictions voluntarily to other advertising, so the government could then claim credit for that also.^55^ At a subsequent meeting in the House of Lords with representatives of the Tobacco Advisory Committee (TAC), the main industry representative organization, the lord president said that the government accepted the scientific case as in the RCP report but was against compulsion and action that would lead to pressure for similar measures in respect to alcohol, and even to foods like chocolate; it was “not the government's purpose to induce any catastrophic change in smoking habits.”^56^ The meeting resulted in a move toward overall agreement on advertising restrictions based on the code applicable to television. On 14 November, Hailsham wrote to Sir Alexander Maxwell, chairman of the TAC and previously wartime Tobacco Controller, that he felt the informal[Other P-303] way this matter had been dealt with was suited to other issues as they arose. But he was clear that he was no stooge for industry interests; someone at Carreras had sent him a box of filter-tipped Piccadilly cigarettes: “This was indeed bearding the lion in his den, but it was as ineffectual as the devil's attempt on St. Anthony.”^57^

The governmental response was thus muted and focused on the strategy of health education. The multiplicity of interests in government was a key factor. The Treasury view ultimately prevailed over the taxation issue, but not before the implications had been fully aired at the political level. The role of the industry was important, although its representatives were called in after the political decisions had been taken. Also behind these decisions was a desire to achieve a balance in policy, and the realization that without a huge change in the social positioning of smoking there was little point in initiating a major program of activity. Discussion of health-education strategies and organization was not the only way in which government considered the implications of the RCP report: the debates about differential taxation and other strategies also led to important developments both in smoking policy and in public health later on, in the 1970s.^58^

But health education was the main response. We can trace the beginnings of the important change in attitude from the 1950s. It was one that also ultimately saw the responsibility for health education move from the local arena to become a national concern. In the late 1950s, at the time of the MRC's statement on smoking and lung cancer, the response had been at the local level through the Medical Officer of Health. The message that came across in public education in the 1950s was equivocal. The idea of outlining specific courses of action was anathema to a society that associated “propaganda” with wartime central direction, and with earlier Nazi propaganda. Health education at this time placed its faith in the citizenship of its recipients. One can see the government departments edging toward this change in the discussion of smoking, prodded also by tensions in the organization and funding of health education. The civil servant Enid Russell Smith, always an incisive analyst of events, commented in 1962 that government could draw in future on two things: parents' concern for their children, and the changes taking place in the medical profession. Publicity would have the authority of the profession. So far, she[Other P-304] commented, the state had not sought to protect individuals from doing harm to their own health if they were not harming the health of others; alcohol was an exception to the rule, and also drugs of addiction, but for both it was the social consequences rather than individual health that was paramount. The new line might be that the costs fell on the state, and so government should stop people from damaging their health—but, she commented presciently, once government took on this role, it would not stop at smoking.^59^ Lung cancer, argued the secretary of state for Scotland, the minister of education, and the minister of health, in an appendix to a policy document prepared just before the 1962 report was published, was a largely preventable disease, but “the question for us is whether it is our duty as a Government to set about preventing it.”^60^

This was the central issue. The period encompassing the end of the 1950s and the beginning of the 1960s was suffused with discussion within government about a reorientation of its role in relation to the health of the public. Although the costs of the newly established NHS were a matter of concern elsewhere in the policy machine, there was no connection between that issue and the potential expansion of the role of government in relation to behavior. Here government was actively resisting its potential new role. It was feared, for example, that more cancer education would lead to greater fear of cancer and hence a greater demand for services, not a reduction. The discussion of the rise in lung cancer was affected by those considerations. The NHS, in any case, was recognized already to be a national *sickness* service rather than a national *health* service, concentrating on disease rather than on positive health. It was not until the 1973 oil crisis that costs and the role of individual behavior began to be considered in tandem. The 1962 report—produced by an “outside” body, not by an offcial committee—brought to a head the issue of whether government should have a role in health behavior, and also highlighted the organizational tensions. It led ultimately, through the Cohen committee report of 1964, to the formation in 1968 of the Health Education Council, a new technocratic central agency responsible for persuasive media campaigns.^61^

## The Control of Advertising

Advertising for health was part of the emergent media and consumerist focus of public health—but advertising was also an activity to be opposed when it was promoting harmful products. The same combination of economics and statistical evidence began to mark governmental activity against tobacco advertising, the other key plank of the government's response to the RCP report. This new consumer strand in policy was symbolized by another report, which arrived in the Ministry of Health just after the publication of the RCP's, from the Advertising Inquiry Council, a body formed in March 1959 in order to represent the interests of the consumer in advertising.^62^ It was a study of expenditure and trends in sales advertising on tobacco, researched and written by an economist and a doctor—a signifcant combination for the future of public health. It looked at the rise in expenditure on tobacco advertising in the early 1960s: advertising costs had risen by 50 percent in one year, 1960, and the public's expenditure on tobacco was also rising. Women's smoking was on the increase, and the teenage market was growing. Filter cigarettes had taken off in popularity in the mid-1950s after their introduction in the late 1940s to save leaf and to save smokers' money after increases in tobacco duty; the report noted that their sales now accounted for 20 percent of the cigarette market. The whole nature of tobacco and cigarette promotion had changed in recent years. The Council's report, which was mentioned in Parliament, added to fears already raised about trends within the tobacco industry: a Monopolies Commission report had drawn attention to its high degree of business concentration, with two firms, Imperial and Gallaher, accounting for over 90 percent of the market. Philip Noel Baker, MP, chairman of the Advertising Inquiry Council, was pressing Macmillan for an advertising ban.^63^ Fletcher and Morris were also involved.^64^ Advertising was an important component of the response to the 1962 report, and the tobacco companies voluntarily offered the removal of all advertising on television before 9 p.m. But concerns later arose on their part about this voluntary concession. Partridge of Imperial[Other P-306] told a Board of Trade official in June 1962 that Imperial and Gallahers had seen advantages in the concession: they had expected to be able to reduce advertising expenditure by 50 percent because of the television restriction—but Rothman Carreras had increased its advertising, and so the manufacturers were beginning to break ranks.^65^ Negotiations about further restrictions dragged on into the 1964 changeover to a Labour government—which took a stronger line.

## The Social Survey, Social Research, and the New Role of the Public

What was also beginning to change in the mid-1960s was the view of “the public” held by politicians and by officials: this was to be a crucial component of future public health initiatives. The commercial techniques of market research expanded in the postwar years and government also began to survey the nature of public opinion and attitudes through the social survey. This surveillance of the population was part of a more general expansion of research and evaluation that was epitomized by the smoking issue. In 1962 a report from the PR frm Armstrong Warden, presented to the Ministry of Health's advisory group on publicity, had pointed out the long-term nature of trying to change public attitudes to smoking. The first job was to convince people that smoking did constitute a danger, and the effects of that should be measured by public opinion research.^66^ As with the change of attitude toward the content of public education, government was edging toward this form of surveillance. A pilot social survey had been carried out in 1960 for the Home Affairs committee by the Social Survey division of the Central Office of Information. This had confirmed the impression given by earlier surveys carried out in Edinburgh to evaluate a campaign led by the MOH there in the 1950s: most of the population was aware of the association between smoking and lung cancer; only one person in the 1960 survey was not, an old lady of eighty-seven who was a nonsmoker. But both the Edinburgh and the pilot surveys had shown that a smaller proportion of the survey population accepted that the association was proved, and a negligible number had given up smoking because of it.^67^

In the mid-1960s the surveillance of public attitudes went further. For the first time, survey research and evaluation accompanied a campaign almost from the start, and research into young people's attitudes to smoking was undertaken. There was also research into medical students' attitudes. The research was carried out by Drs. Aubrey McKennell and R. K. Thomas of the Social Survey division and by the social psychologist John Bynner. Bynner's work on adolescent smoking was based on the smoking questions in a wartime survey of adolescent sexual behavior by the Central Council for Health Education.^68^ The results of the McKennell survey, started in 1963 and first reported to the officials' committee in 1964 when the American surgeon general's report was under consideration, emphasized the potential new role for government health education: “The ethics or appropriateness of using such an approach in Government publicity needs to be faced. The use of somewhat devious, emotional rather than straightforward means of persuasion is of course, for better or worse, a characteristic of much successful commercial advertising.”^69^

Other survey research was carried out by social scientists and epidemiologists, and increasingly this focused on the young. The sociologist Margot Jefferys was involved in the 1950s and early 1960s in a study of Harlow New Town with other researchers from the London School of Hygiene; her study of the impact of health education on children's attitudes toward smoking was one of the first academic publications in the feld.^70^ The choice of smoking and of children also indicated the reorientation of this type of “community study,” which had until then concentrated on the environment rather than individual issues. Jefferys, as a key fgure in the Society for Social Medicine in this period, was part too of the transformation of social medicine into a new form of public health that the smoking work symbolized.^71^ In the discussions of the ongoing research [Other P-308] in the Central Offce of Information and the Ministry of Health can be seen in embryo the emergent evaluative paradigm of “relevant research,” a precursor of later evidence-based tendencies in health research.^72^

## The Electoral Argument Diminishes

The publication of the American surgeon general's report in 1964 led to a further officials' report and to political interest. The American report extended associations between smoking and health risk to diseases other than lung cancer, but British offcials did not feel that this warranted further action. On 30 June 1964 the cabinet committee approved the officials' suggestion of a modest extension of the government's health-education campaign. There was no support for a ban on TV advertising or on smoking in cinemas. Least opposition was attracted by packet warnings. Lord Hailsham wanted more action. On 6 April 1964, he wrote in response to his officials' lack of enthusiasm: “I consider that the American Report, the American action and the Social Survey *have* strengthened the case for action, and that it is *not* too early to say that our limited campaign is failing and that unless we can bare our teeth nothing that we do *will* be taken seriously.”^73^ He also inserted a signifcant change in the inequality argument deployed by officials: the words “it would bear more hardly on the poor than on the rich” were replaced by “it could be harder for a poor man than for a rich man to continue his existing level of smoking and while this element of discrimination might be said to be more to the poor man's beneft, it would be unlikely to go uncriticised.”^74^ But Hailsham's response in 1964 was unusual for the time. As he pointed out in the Commons adjournment debate on the surgeon general's report, he was a nonsmoker in a Parliament of smokers, a cabinet of smokers, and an electorate of smokers. His views did not at that stage represent either the cultural or the political norm.^75^

In 1961, Enoch Powell, the Conservative minister of health when the RCP report was published, had expressed his opposition to media [Other P-309]strategies.^76^ In an interview conducted in 1975, Powell was more forthcoming about the roots of his opposition. Governments did not like to reorganize taxation, and then there was the question of harm, which, in the case of smoking, was fluid and vague. Legislating against a widespread and common form of behavior was very different from legislating against an uncommon and marginal form. Governments would be very foolish to act without overwhelming evidence—and here the 1962 report, in his view, did make a difference to the clarity of the issue.^77^

Kenneth Robinson, as Labour minister of health in the mid- to late 1960s, was more active against smoking, but his view of policy was also that it was constrained by public opinion, not by financial considerations. He also stressed that the main constraint on government had been that there was no public support for action against smoking. The answer, as he saw it, was to change the climate of opinion through health education, in particular with themes like smell and attractiveness that appealed to young people.^78^ The Labour politician Richard Crossman's opposition to Robinson's proposed changes in smoking policy in the later 1960s was also prompted by electoral considerations.^79^ But this argument began to change in the 1970s when Conservative politicians like Keith Joseph and Labour politicians like David Owen and Dennis Healey as chancellor of the exchequer saw dawning electoral advantage in antismoking measures.

## Conclusion

The RCP report of 1962 was the forerunner of later College reports on smoking and a host of other health-related subjects, all of which were aimed at both government and the public. The “medical voice” developed important relationships with both government and the public in areas that would not previously have been considered the province of either. In the 1970s this insider/outsider relationship for medicine developed further into a host of expert committees with close relationships within government. The RCP report in 1962 was a signifcant stage in moves toward a new era in which the presentation of science to the public through the [Other P-310] media, with the authority of scientists and the medical profession, became central. As consumerist trends in society consolidated, and medicine and public health both sought “modernization,” the old tradition of “giving the facts” to citizens was transformed into warnings about health risk. The nature of public opinion and “the public” was exposed to research-based surveillance. The techniques of social as well as medical science were brought into play. These changes were recognizably rooted in some of the postwar transformations of social medicine, but they also incorporated new commercial techniques of persuasion and commercial ideas about research. The permissive-society analysts have argued for a diminished state role for some health-related issues—but the case of smoking, and the new ideas within public health, show the infuence of the state as increasing, not diminishing. This infuence was exerted through new relationships with the medical profession and with research, and through new agencies. Government and the medical profession began to share a belief in the power of the media and of advertising to alter public attitudes.^80^ This is what I have called “coercive permissiveness”: members of the public could modify their own habits and lifestyle to gain better health, but increasingly that modifcation was state ordained and supported. The case of smoking and the RCP report shows how such ideas and interests were beginning to shape a distinctive postwar British public health ideology, separate from the organizational base of the profession in health services and community medicine that has attracted most commentary. The report mediated between social medicine in fux and the new evidence-based medicine and public health.
